# Prevalence and Risk Factors of Pressure Ulcers in Bedbound Diabetic vs. Non-diabetic Patients in a Lower-Middle-Income Country

**DOI:** 10.7759/cureus.61861

**Published:** 2024-06-06

**Authors:** Diyan Muhammad, Khola Darain, Muhammad Farhan, Muhammad Anas Khan, Hussain Ahmad, Muhammad Mohsin Khan, Zia Ullah, Sibghat Ullah, Abdus Salam, Shakir Ullah, Junaid Khan, Muhammad Zarin

**Affiliations:** 1 General Surgery, Khyber Teaching Hospital, Peshawar, PAK; 2 General Surgery, Lady Reading Hospital, Peshawar, PAK; 3 Internal Medicine, Hayatabad Medical Complex Peshawar, Peshawar, PAK; 4 Oncology, Hayatabad Medical Complex Peshawar, Peshawar, PAK; 5 General Surgery, Hayatabad Medical Complex Peshawar, Peshawar, PAK; 6 Orthopedics, Khyber Teaching Hospital, Peshawar, PAK

**Keywords:** pakistan, cross sectional studies, pressure sores, hospitalized patients, bedridden, decubitus ulcers, caregiver, immobilized, diabetics, pressure ulcers

## Abstract

Introduction

Pressure ulcers, also known as bedsores, are a significant concern for bedridden individuals, presenting both physical and socioeconomic challenges. Factors such as prolonged immobility, chronic medical conditions, and poor nutrition contribute to their development. Despite extensive research in some regions, studies comparing diabetic and non-diabetic populations remain limited, particularly in low-income settings. This study aimed to investigate the risk factors and frequency of pressure ulcers among bedridden patients, addressing this gap in understanding and guiding targeted interventions.

Materials and methods

A cross-sectional study was conducted across four government hospitals in Peshawar, Pakistan. A total of 388 bedridden patients with pressure ulcers were included, and data were collected through a questionnaire. The questionnaire covered demographics, comorbidities, duration of bedbound status, BMI, and caregivers’ awareness of pressure ulcer care. Data analysis was performed using SPSS version 22.0 (Armonk, NY: IBM Corp.), with qualitative data presented as frequencies and percentages and quantitative data as mean and standard deviation. Chi-square tests were utilized for significance, with p<0.05 considered significant.

Results

Of the 388 patients analyzed, 230 (59.3%) were diabetic, highlighting the prevalence of diabetes among pressure ulcer cases. The majority of diabetic patients with ulcers were over 41 years old, and 293 (75.5%) had comorbidities. Surgical intervention was the primary cause of ulcers in 213 (54.8%) cases, followed by stroke in 77 (19.8%) cases. Notably, 252 (65%) of caregivers exhibited inadequate knowledge regarding ulcer care. Stage II ulcers were prevalent in both diabetic and non-diabetic cohorts.

Conclusions

Pressure ulcers are poorly controlled complications observed in bedridden individuals, highlighting a critical need for comprehensive preventive measures and caregiver education to alleviate the burden of pressure ulcers, especially in diabetic patients. Factors such as prolonged immobility, surgical interventions, and insufficient caregiver knowledge contribute to the development of pressure ulcers. Understanding these complexities is essential for implementing effective care approaches and mitigating the impact of pressure ulcers.

## Introduction

Bedsores are also called pressure ulcers or decubitus ulcers. Pressure sores are areas of localized damage to the skin or underlying tissue or both due to pressure, shear, friction, or maybe all of these [1]. In bedridden patients, one of the main issues is the development of pressure sores. Chronic wounds like bedsores can be a problem biologically, socially, psychologically, and financially for healthcare workers, attendants, and patients [2]. Patients whose mobility is restricted due to any medical or surgical issue usually develop pressure ulcers [3,4]. Thus, unrelieved pressure overrides capillary pressure leading to tissue ischemia and ultimately necrosis and chronic wounds and ulcers. The skin over bony prominences like sacrum, hip, and malleoli bones are particularly notorious for their vulnerability to as little as two hours of immobility, though other sites can be affected, such as the elbows, knees, ankles, back of shoulders, or the back of the cranium [5]. Diabetes mellitus with over 382 million patients all over the world is notorious for non-healing wounds as background, the most common and serious of which is diabetic foot. A triad of neuropathy, ischemia, and trauma is known to be the pathophysiology [6].

As per the literature, pressure ulcers are a major problem in healthcare facilities and the general bedridden population, influenced by other factors such as age and BMI, chronic medical issues, decreased mobilization, and peripheral vascular diseases [7]. Additionally, poor nutrition, co-morbidities, male gender, and prolonged bed rest, further escalate the risk [3]. All bedridden patients should be aware of pressure ulcer prevention, proper positioning, nutrition, and skincare. Also, skin moisturization, proper nutrition, patient education, reduction of pressures on bony prominences and dependent areas, and periodic mobilization of patients can reduce the rate of pressure ulcers significantly.

Although preventable in most cases, pressure ulcers continue to be a major burden to the individual and society, affecting ≤3 million adults annually in the United States alone [2]. It is a major healthcare problem costing billions of dollars each year in the United States and even more in the developing world. Despite increased national attention over the past 20 years, the prevalence of pressure ulcers has remained largely unchanged, while the associated costs of care continue to rise [8].

For every unit million patients who developed bedsores, 65,000 died of complications, making it a major problem across the globe [1]. As literature revealed, bedsores are common in middle- and high-income parts of the world and are rarely researched in low-income countries, such as a prevalence of 12.7% was reported in Brazil, 10.4% in Turkey, 47.6% in Thailand, and 16% in Ethiopia [1]. Since there is a risk of bedsores in any bedridden or compromised patients and certain factors affecting it, still this topic is rarely studied in comparison between diabetic and non-diabetic patients as per the literature review, and no local studies were found. We initiated this study with the aim of identifying potential risk factors and the frequency of bedsores in bedridden patients, as there may be correlations between various factors in both diabetic and non-diabetic individuals.

## Materials and methods

Objectives

This study aimed to assess the variance in the prevalence of pressure ulcers between diabetic and non-diabetic bedbound patients in the mentioned settings, to analyze the disparity in the occurrence of pressure ulcers between male and female bedbound patients in the mentioned settings, to investigate the influence of body mass index (BMI) on the incidence of pressure ulcers among bedbound patients, stratified by diabetic and non-diabetic status, in the mentioned settings, and to explore the impact of patient and caregiver awareness regarding pressure ulcers on the frequency of occurrence in the mentioned settings.

Study design and settings

This cross-sectional study was conducted in four hospitals - Khyber Teaching Hospital, Peshawar, Hayatabad Medical Complex Peshawar, Lady Reading Hospital, Peshawar, and Paraplegic Center, Hayatabad, Peshawar.

Target population and sample size

All diabetic and non-diabetic patients presenting to Khyber Teaching Hospital, Hayatabad, Medical Complex, Lady Reading Hospital, and Paraplegic Center, Hayatabad, Peshawar, were the target population for this study. The sample size was calculated using a sample size calculator, assuming a prevalence of 50% of pressure sores in all bedbound patients in Pakistan, with a total population of unlimited size. With a 95% confidence interval and a precision of ±5%, the estimated minimum sample size calculated was found to be 388.

Sampling technique

In our study, we utilized purposive or judgmental sampling to select bedbound patients with and without diabetes. This approach ensured the relevance of our sample for examining pressure ulcer risk factors and frequency in these specific populations.

Inclusion and exclusion criteria

All diabetic and non-diabetic patients presenting to the hospital with pressure ulcers of any stage, who had been bedridden for any cause for at least one week or more were included in this study. All patients who did not give consent were excluded from this study.

Data collection procedure

Data were collected by means of a questionnaire that was filled out either by the primary caregivers of patients who accompanied them to the hospital or by the interviewer if the primary caregiver was unable to read or write. Written informed consent was obtained from every patient. If consent was not given, the respondent was not included in the study, and their refusal was noted without making a second attempt to obtain consent.

Data collection tool

A standardized questionnaire was developed for data collection. It was available in English and translated into Pashto or Urdu by the interviewer, where necessary. The questionnaire was validated and readjusted after a pilot study was conducted on 25 participants. The questionnaire included two sections. The first section included 11 questions about patients' demographics, comorbidities, duration and causes of being bedbound, and their knowledge about their primary disease and pressure ulcers. The second section of the questionnaire included six questions for the primary caregiver regarding their knowledge of the disease, particularly pressure ulcer care.

Data analysis

The data were entered into SPSS version 22.0 (Armonk, NY: IBM Corp.) and manually reviewed for discrepancies and missing data. All variables were then coded for analysis. The Chi-square test was used to determine significance, with a p-value of less than 0.05 considered significant.

Ethical considerations

This study was approved by the Institutional Research and Ethical Review Board of Khyber Medical College (IRB number: 498/DME/KMC). All respondents participating in this study were assured of complete confidentiality, and their responses were kept anonymous, with access restricted to the principal research team. All individuals involved in this project had the right to withdraw from participation at any point in time. No incentives were offered to participants for their involvement in the study.

## Results

In our study, we analyzed 388 patients with bedsores, comprising 230 (59.3%) diabetic individuals and 158 (40.7%) non-diabetic individuals. As shown in Table 1, a remarkable number of caregivers lack sufficient knowledge about caring for and preventing pressure ulcers, accounting for 107 (27.6%) with no knowledge and 145 (37.4%) with some knowledge, while only a few have sufficient knowledge, totaling 6 (1.5%).

**Table 1:** The frequency distribution of different variables of patients having pressure ulcers with comparison of diabetic and non-diabetic status. n: sample size

Variables		Diabetic n=230 (59.3%)	Non-diabetic n=158 (40.7%)	Total n=388	p- Value
Gender	Male	111 (54.1%)	94 (45.8%)	205 (52.8%)	0.005
	Female	119 (65.1%)	64 (34.9%)	183 (47.2%)	
Demographics	Rural	158 (67.2%)	77 (32.8%)	235 (60.5%)	0.029
	Urban	72 (47.0%)	81 (53.0%)	153 (39.5%)	
Comorbidities	Yes	179 (61.9%)	114 (38.1%)	293 (75.5%)	0.271
	No	52 (54.7%)	43 (45.3%)	95 (24.5%)	
Body mass index (BMI) status	Underweight	42 (31.6%)	91 (69.4%)	133 (34.27%)	0.001
	Normal	68 (70.1%)	29 (29.9%)	97 (25%)	
	Obese class 1	81 (77.1%)	24 (22.9%)	105 (27.1%)	
	Obese class 2	41 (77.4%)	12 (22.6%)	53 (13.7%)	
Education level of caregivers	No education	36 (53.8%)	31 (46.2%)	67 (17.3%)	0.153
	Undergraduate	135 (63.4%)	78 (36.6%)	213 (54.9%)	
	Graduate	59 (54.7%)	50 (46.3%)	108 (27.8%)	
Awareness of caregivers regarding pressure ulcers	No knowledge	67 (62.7%)	40 (37.3%)	107 (27.6%)	0.021
	Somewhat	98 (67.6%)	47 (32.4%)	145 (37.4%)	
	Most of it	48 (47.6%)	53 (52.4%)	101 (26%)	
	Almost enough	15 (51.7%)	14 (48.3%)	29 (7.4%)	
	Everything	2 (33.3%)	4 (66.7%)	6 (1.5%)	

**Figure 1:**
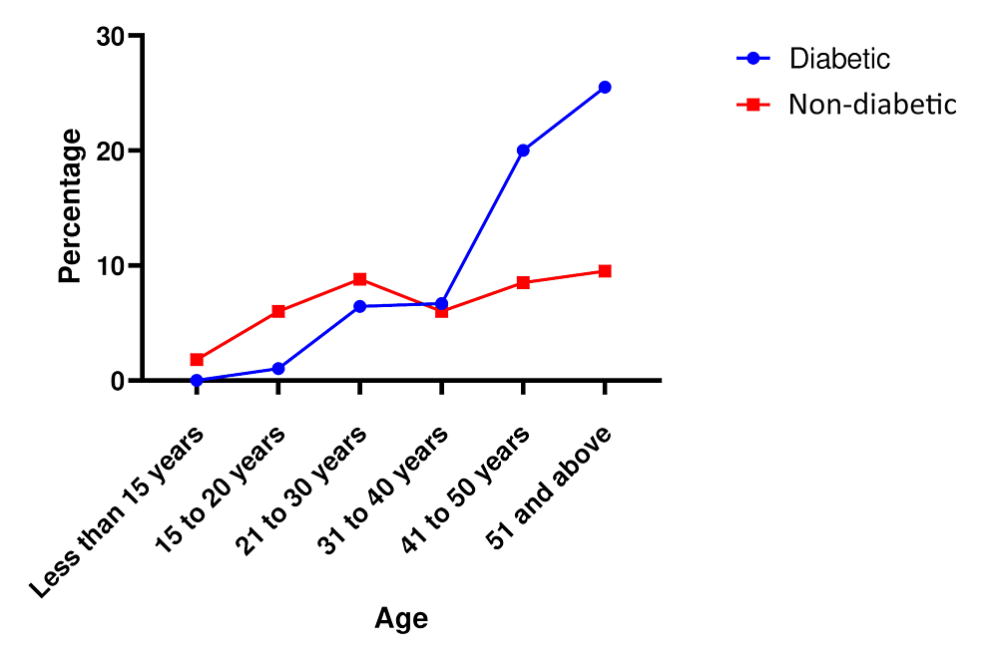
Pressure ulcers in diabetics vs. non-diabetics in different age groups.

**Figure 2:**
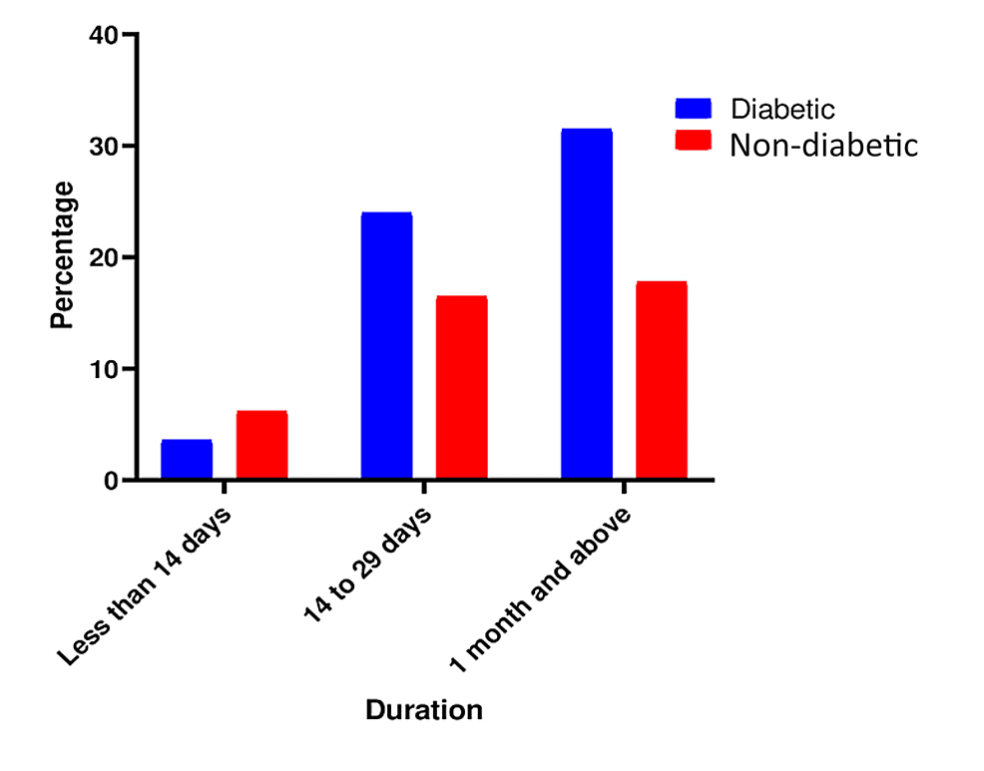
Percentage of pressure ulcers in relation to bedbound duration.

Among diabetic patients with pressure ulcers, 294 (75.8%) were older than 41 years, indicating that age plays an important role in the development of pressure ulcers and is directly proportional to it (Figure 1). Of the total participants, 205 (52.8%) were male, and the remaining were female, indicating that male gender is an important predisposition. In rural areas, pressure ulcers are more prevalent, with 235 (60.5%) cases compared to urban areas. Additionally, a higher percentage of rural residents (158 {67.2%}) with pressure ulcers have diabetes compared to urban residents (72 {47%}). The majority of patients, accounting for 293 (75.5%), presented with comorbidities, proving that other co-morbidities along with diabetes exacerbate the chances of pressure sores. Furthermore, 191 (49.2%) patients had been bedridden for more than a month (Figure 2).

**Figure 3:**
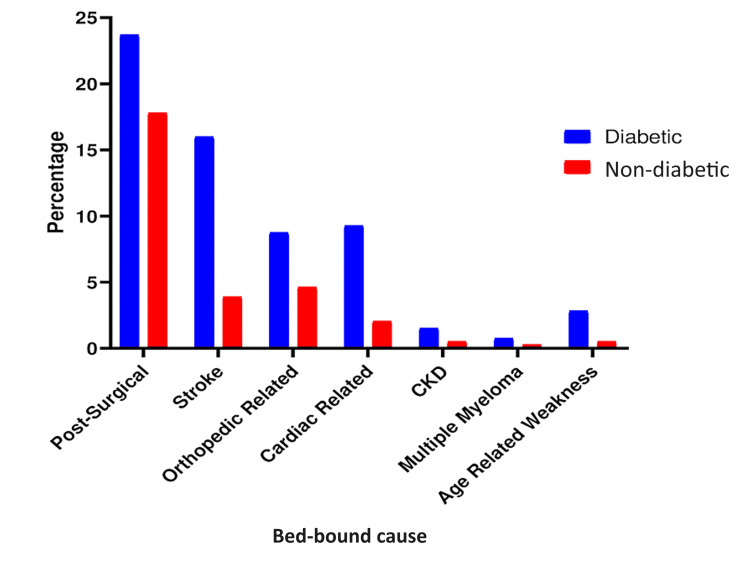
Percentage of pressure ulcers and primary cause of being bedbound. CKD: chronic kidney disease

**Figure 4:**
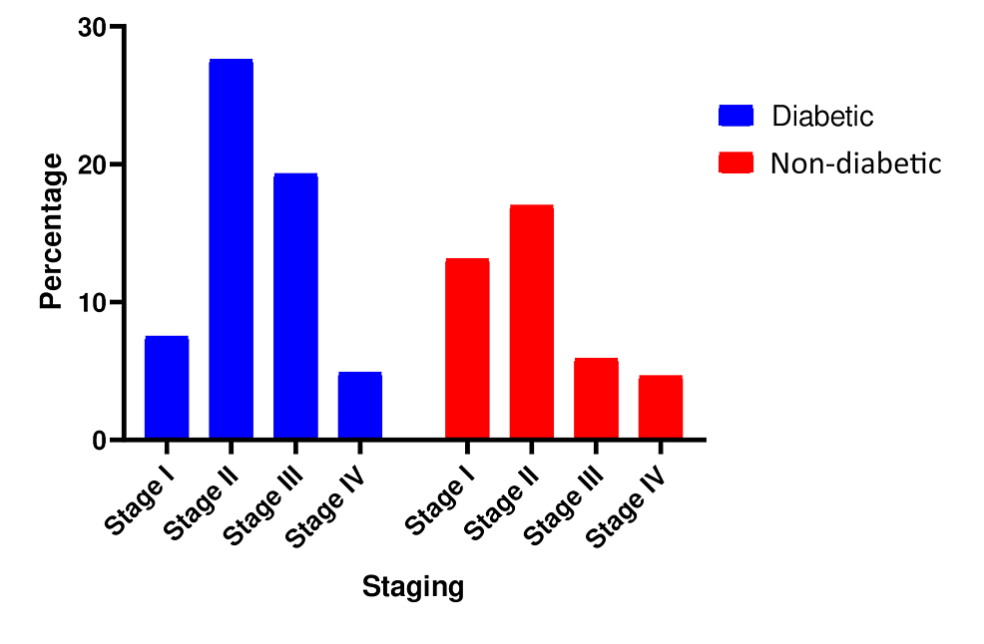
Percentage of stages of pressure ulcers during presentation.

The primary cause of bedsores in our study was surgical intervention, with 213 patients (54.8%) experiencing bedsores as a complication, followed by stroke, which accounted for 77 (19.8%) patients (Figure 3). Figure 4 reveals that the majority of patients, 173 (44.5%), had stage 2 pressure ulcers, while a small number of patients, 37 (9.5%), had stage 4 ulcers, indicating that most patients present to the hospital with stage 2 or higher stages of pressure ulcers.

## Discussion

Pressure ulcers are poorly controlled complications seen in bedridden individuals. According to the recent definitions issued by the National Pressure Ulcer Advisory Panel (NPUAP), the term "pressure ulcer" was replaced by the term "pressure injury" in the National Pressure Ulcer Advisory Panel Pressure Injury Staging System. According to the NPUAP, the change in terminology was made as it accurately describes pressure injuries to both intact and ulcerated skin. According to NPUAP, a pressure injury is defined as localized damage to the skin and/or underlying soft tissue, usually over a bony prominence or related to a medical or other device. This injury can present as intact skin or as an open ulcer and may or may not be painful. The injury occurs as a result of intense and prolonged pressure or pressure in combination with shear. The tolerance of soft tissue for pressure and shear may also be affected by microclimate, nutrition, perfusion, comorbidities, and the condition of the soft tissue [9].

Our investigations delve into the profound differences between pressure ulcers in diabetic vs. non-diabetic patients. Involving data from multiple hospitals, our study illuminates an evident reality that diabetic patients comprise 230 (59.3%) pressure ulcer cases. This prevalence underscores the critical need to understand the complexity of diabetic care to lessen the risk of pressure ulcers, a finding consistent with previous research [10-12]. Diabetes mellitus not only indicates a poor prognosis but also increases the risk of pressure sore development due to impaired healing mechanisms inherent to diabetes [8].

Diabetic patients are particularly vulnerable to pressure ulcers and face an increased risk, especially if confined to bed for seven days or more [7]. This correlation between extended hospital stays and pressure ulcer incidence resonates with evidence-based studies, emphasizing the dangers of prolonged immobility and increased contact time with pressure ulcers [3]. Obese patients exhibit a higher incidence of pressure ulcers compared to those with a normal BMI, as evident in a 2019 study by Mervis and Phillips [8]. This correlation is also verified in a meta-analysis in 2023 [3]. Our investigations affirm that pressure ulcer staging rises with prolonged bed rest, supported by a study conducted in Ethiopia [1].

Our study reveals that a staggering 252 (65.0%) caregivers exhibit inadequate knowledge regarding diabetic and pressure ulcer care and prevention. This coincides with findings from Ethiopia, underscoring the important role of caregiver education in reducing the burden of pressure ulcers [13]. Post-surgical patients face a significant risk, with 161 (41.5%) experiencing pressure ulcers post-intervention. This burden increases dramatically in the presence of diabetes, as confirmed by a comprehensive meta-analysis conducted in 2015 [14]. Stage II pressure ulcers, prevalent in both diabetic and non-diabetic patients, manifest excruciating pain owing to intact sensory nerves extending into the dermis, serving as a bitter reminder of the debilitating consequences of this condition [8].

Overall, the study provides valuable insights into the complex interplay between diabetes, immobility, caregiver awareness, surgical interventions, and pressure ulcer development, highlighting the importance of comprehensive preventive measures and care approaches.

Limitations

Our study does not capture all relevant risk factors for pressure ulcer development, such as nutritional status, socioeconomic factors, compliance with comorbidities such as controlled or uncontrolled glycemic levels or hypertension, etc., which could have significant implications for preventive strategies.

The cross-sectional nature of our study limits the ability to establish temporal relationships between risk factors and the development of pressure ulcers. Longitudinal studies would provide stronger evidence of causality. By addressing these limitations in future studies and interventions, we can improve the understanding and management of pressure ulcers, ultimately enhancing patient care and outcomes.

## Conclusions

Pressure ulcers are poorly controlled complications seen in bedridden individuals, and there is a critical need for comprehensive preventive measures and caregiver education to reduce the burden of pressure ulcers, particularly in diabetic patients. Factors such as prolonged immobility, surgical interventions, and inadequate caregiver knowledge contribute to the development of pressure ulcers. Understanding these complexities is essential for implementing effective care approaches and reducing the impact of pressure ulcers.
